# Activation of the ARC^POMC^→MeA Projection Reduces Food Intake

**DOI:** 10.3389/fncir.2020.595783

**Published:** 2020-11-05

**Authors:** Eunjin Kwon, Young-Hwan Jo

**Affiliations:** ^1^The Fleischer Institute for Diabetes and Metabolism, Bronx, NY, United States; ^2^Division of Endocrinology, Department of Medicine, Bronx, NY, United States; ^3^Department of Molecular Pharmacology, Albert Einstein College of Medicine, Bronx, NY, United States

**Keywords:** melanocortin, estrogen, stress, POMC (proopiomelanocortin), anorexia, amygdala

## Abstract

Proopiomelanocortin (POMC) neurons in the arcuate nucleus of the hypothalamus (ARC) plays an essential role in the control of food intake and energy expenditure. Melanocortin-4 receptors (MC4Rs) are expressed in key areas that are implicated in regulating energy homeostasis. Although the importance of MC4Rs in the paraventricular hypothalamus (PVH) has been well documented, the role of MC4Rs in the medial amygdala (MeA) on feeding remains controversial. In this study, we specifically examine the role of a novel ARC^POMC^→MeA neural circuit in the regulation of short-term food intake. To map a local melanocortinergic neural circuit, we use monosynaptic anterograde as well as retrograde viral tracers and perform double immunohistochemistry to determine the identity of the neurons receiving synaptic input from POMC neurons in the ARC. To investigate the role of the ARC^POMC^→MeA projection on feeding, we optogenetically stimulate channelrhodopsin-2 (ChR2)-expressing POMC fibers in the MeA. Anterograde viral tracing studies reveal that ARC POMC neurons send axonal projections to estrogen receptor-α (ER-α)- and MC4R-expressing neurons in the MeA. Retrograde viral tracing experiments show that the neurons projecting to the MeA is located mainly in the lateral part of the ARC. Optogenetic stimulation of the ARC^POMC^→MeA pathway reduces short-term food intake. This anorectic effect is blocked by treatment with the MC4R antagonist SHU9119. In addition to the melanocortinergic local circuits within the hypothalamus, this extrahypothalamic ARC^POMC^→MeA neural circuit would play a role in regulating short-term food intake.

## Introduction

Melanocortin-4 receptors (MC4Rs) play a critical role in regulating food intake and energy expenditure and in preventing obesity (Huszar et al., 1997; Balthasar et al., 2005; Rossi et al., 2011; Liu et al., 2013; Berglund et al., 2014; Shah et al., 2014). MC4Rs are expressed throughout the brain (Kishi et al., 2003), including several areas that are implicated in the control of energy balance (Kishi et al., 2003; Liu et al., 2003). In particular, MC4Rs in the paraventricular hypothalamus (PVH) appear to be responsible for melanocortin-mediated regulation of food intake (Balthasar et al., 2005). Selective restoration of MC4Rs in single-minded 1 (SIM1) neurons in mice lacking MC4Rs (i.e., *Sim-Cre*; loxTB *Mc4r* mice) results in significantly reduced body weight gain and food intake, compared with loxTB *Mc4r* mice (Balthasar et al., 2005). Detailed neuroanatomical studies further demonstrate that MC4R-expressing SIM1 neurons in the PVH, but not the medial amygdala (MeA), are necessary for MC4R-mediated anorectic effects (Shah et al., 2014). Additionally, it has been shown that these MC4R-expressing neurons are glutamatergic and send projections to the lateral parabrachial nucleus (PBN; Shah et al., 2014), an area that mediates the suppression of appetite (Carter et al., 2013; Garfield et al., 2015). Thus, MC4R-expressing glutamatergic neurons in the PVH and the neurons in the PBN appear to be a functionally important circuit for regulating food intake.

Outside the hypothalamus, the MeA that controls emotional behaviors, including stress-induced anxiety (Liu et al., 2013) and male social behavior (Cushing et al., 2008; Sano et al., 2016) highly expresses MC4Rs (Kishi et al., 2003; Liu et al., 2003). Importantly, it has been shown that MC4Rs in the MeA regulate feeding (Liu et al., 2013). For instance, local infusion of a selective MC4R agonist into the MeA decreases food intake in rodents (Liu et al., 2013). Early studies demonstrate the presence of melanocortinergic input to the MeA (Jacobowitz and O’Donohue, 1978; Watson et al., 1978). The central melanocortin system such as pro-opiomelanocortin (POMC) neurons and agouti-related peptide (AgRP)/neuropeptide Y (NPY) neurons in the arcuate nucleus of the hypothalamus (ARC) appears to project to the MeA (Jacobowitz and O’Donohue, 1978; Padilla et al., 2016). In particular, AgRP neurons send inhibitory input to NPY 1 receptor (NPY1R)-expressing neurons, and activation of NPY1R-expressing neurons in the MeA reduces food intake (Padilla et al., 2016). Therefore, both the MeA and the PVH would be downstream targets of the central melanocortin system.

The MeA contains estrogen receptor-α (ER-α)-expressing cells (Xu et al., 2015; Saito et al., 2016). Importantly, it has been well described that estrogen receptor-α (ER-α) activity in the MeA controls energy balance as well (Liu et al., 2013; Estrada et al., 2018). For example, local implantation of estradiol in the MeA or treatment with estradiol reduces food intake, body weight, and adiposity observed in ovariectomized female rodents (Xu et al., 2017; Estrada et al., 2018). Specific deletion of ER-α in SIM1 neurons in the MeA causes obesity in both male and female mice, while overexpression of ER-α prevents diet-induced obesity (DIO) in male mice (Xu et al., 2015). As the MeA receives dense α-melanocyte-stimulating hormone (α-MSH)-positive fibers (Jacobowitz and O’Donohue, 1978) and SIM1 neurons in the MeA express both ER-α and MC4Rs (Balthasar et al., 2005; Xu et al., 2015), we examined the role of this ARC^POMC^→MeA projection in the control of feeding. Antero- and retrograde viral tracing studies revealed that ARC POMC neurons sent direct axonal projections to the MeA. In particular, ER-α- and MC4R-expressing neurons in the MeA were innervated by ARC POMC neurons. Optogenetic stimulation of this ARC^POMC^→MeA projection reduced short-term food intake. Therefore, the ARC^POMC^→MeA pathway that we identified would be an important melanocortinergic circuit for regulating feeding.

## Materials and Methods

### Ethics Statement

All mouse care and experimental procedures were approved by the Institutional Animal Care Research Advisory Committee of the Albert Einstein College of Medicine and were performed following the guidelines described in the NIH guide for the care and use of laboratory animals. Stereotaxic surgery and viral injections were performed under isoflurane anesthesia.

### Animals

Mice used in this study included POMC-Cre (Stock #005965), floxed-stop GFP (Stock #004077), and floxed-stop ChR2-tdTomato (Stock #012567) transgenic mice (The Jackson Laboratory). Both female and male mice of mixed C57BL/6J, FVB, and 129 strain backgrounds were used. Animals were housed in groups in cages under conditions of controlled temperature (22°C) with a 12:12 h light-dark cycle and fed a standard chow diet with *ad libitum* access to water.

### Stereotaxic Surgery and Viral Injections

Six to seven-week-old mice were anesthetized deeply with 3% isoflurane. A deep level of anesthesia was maintained throughout the surgical procedure. Under isoflurane anesthesia (2%), a Cre-inducible anterograde viral tracer AAV1.CAG.FLEX.GFPsm_myc.WPRE.SV40 (AAV1-Flex-GFPsm; UPenn Vector core; Viswanathan et al., 2015) was bilaterally injected to the ARC of POMC-Cre mice (AP, −1.75 mm, ML, ±0.15 mm, DV, −5.8 mm; 150 nl of 1.36 × 10^13^ pfu/ml per side, *n* = 6 mice). This viral tracer has been shown to label long-range axonal projections (Viswanathan et al., 2015), which permits the mapping of neural circuits. We also used a Cre-dependent retrograde virus retroAAV.CAG.FLEX.tdTomato (retroAAV-FLEX-tdTomato, Addgene). This viral vector was unilaterally injected to the MeA (AP, −1.58 mm; ML, −2.0 mm; DV, −5.0 mm; 200 nl of 1.3 × 10^13^ pfu/ml, *n* = 8 mice). Animals were sacrificed and perfused at 3-4 weeks post-viral injections to perform immunohistochemistry.

### Optogenetic Stimulation of the ARC^POMC^→MeA Projection

We crossbred the POMC-Cre strain with the floxed-stop ChR2-tdTomato strain to generate POMC-Cre; ChR2-tdTomato mice. To stimulate the ARC^POMC^→MeA projection, a mono fiber-optic cannula of 230 μm diameter (MFC_200/230–0.48_5 mm, Doric Lenses) was implanted into the MeA (AP, −1.58 mm; ML, −2.0 mm; DV, −5.0 mm) and coupled to a 473 nm diode-pumped solid-state (DPSS) laser (Laserglow Technologies). Animals were allowed at least a week to recover from surgery. ChR2-expressing POMC fibers in the MeA were illuminated at 20 Hz (25 ms pulse duration, 20 Hz pulses per 1 s, 3 s interval between events). Stimulation pulses were generated with Doric Neuroscience Studio software (Doric Lenses). Light illumination started 30 min before food presentation. To measure liquid food intake (Ensure Plus, Abbott), animals were separated individually in single cages for 5 days and fasted for 6 h (from 12 PM to 6 PM). The MC4R antagonist SHU-9119 (1 mg/kg) was intraperitoneally injected 30 min before food presentation.

### Immunofluorescence Staining

Mice were anesthetized with isoflurane (3%) and transcardially perfused with the pre-perfusion solution (9 g NaCl, 5 g sodium nitrate, 10,000 U heparin in 1 L distilled water). Brains were post-fixed in 4% paraformaldehyde overnight at cold room and sectioned with a vibratome in 40 μm on the following day. The sections were blocked in 0.1 M PBS buffer containing 0.2 M glycine, 0.1% Triton X-100, 10% normal donkey serum, and 5% bovine serum albumin for 2 h at room temperature and then incubated with mouse anti-GFP (1:200, Invitrogen, cat# A-11120), rabbit anti-POMC (1:1,000, Phoenix pharmaceuticals, cat# H-029-30), mouse anti-ACTH (1:100, Santa Cruz, cat# sc-57021), sheep anti-α-MSH (1:10,000, Millipore, cat# AB5087), rabbit anti-MC4R (1:250, Alomone labs, cat# AMR-024), and rabbit anti-ER-α (1:40,000, Millipore, cat# 06-935) antibodies for 72 h at cold room. The sections were washed three times in PBS and incubated with Alexa 488 anti-mouse IgG (1:200; Invitrogen, cat# A11001), Alexa 568 anti-rabbit IgG (1:500, Life Technologies, cat# A10042), or Alexa 568 anti-mouse IgG (1:500, Life Technologies, cat# A10037) for 2 h at room temperature. For co-labeling of ER-α and MC4Rs, we used an Alexa 488 conjugated MC4R antibody (1:200, G-Biosciences, cat#ITA 5860) as both ER-α and MC4R antibodies are from the same species. Tissues were washed, dried, and mounted with VECTASHIELD media containing DAPI. Images were acquired using a Leica SP8 confocal microscope.

### Statistics

All statistical results are presented as mean ± SEM. Statistical analyses were performed using Graphpad Prism 7.0. Two-tailed Student’s *t*-tests were used to calculate *p-values* of pair-wise comparisons. Data for comparisons across more than two groups were analyzed using a one-way ANOVA with *post hoc* Tukey’s multiple comparisons. Data were considered significantly different when the probability value was less than 0.05.

## Results

### ARC POMC Neurons Send Axonal Projections to the MeA

**Figure 1:**
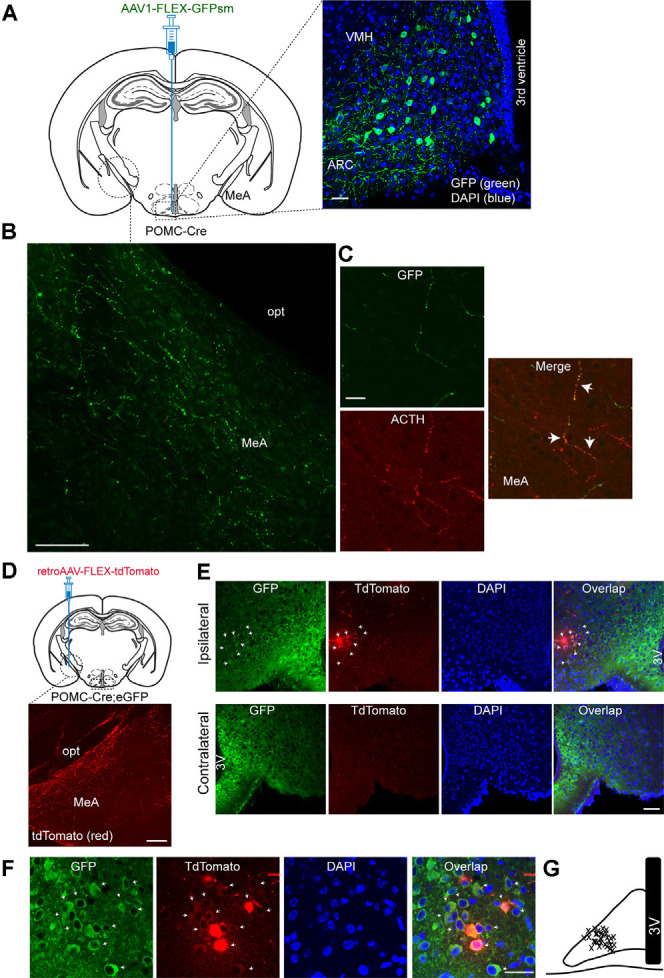
Proopiomelanocortin neurons in the arcuate nucleus of the hypothalamus project to the medial amygdala (MeA). **(A)** Schematic illustration showing our experimental configurations (left panel). A Cre recombinase-inducible anterograde viral tracer AAV1-FLEX-GFPsm was injected into the ARC of the POMC-Cre mice. Right panel: The ARC of the POMC-Cre mice injected with AAV1-FLEX-GFPsm exhibited GFP-positive cells in the ARC. Scale bar: 30 μm. **(B)** Image of fluorescence confocal microscopy showing GFP-positive fibers and terminals in the MeA of the POMC-Cre mice injected with AAV1-FLEX-GFPsm. opt, optic tract, Scale bar: 100 μm. **(C)** Images of fluorescence confocal microscopy showing co-expression of GFP and ACTH in axonal fibers in the MeA (white arrow). Scale bar: 20 μm. **(D)** Schematic illustration showing our experimental configurations (top panel). POMC-Cre; eGFP mice were stereotaxically injected with a Cre-inducible retrograde viral tracer retroAAV-FLEX-tdTomato into the MeA. TdTomato-positive fibers were observed in the MeA (bottom panel). Scale bar: 200 μm. **(E)** Images of fluorescence confocal microscopy showing retrogradely identified ARC POMC neurons projecting to the MeA. Retrogradely identified cells (white arrow) were found in the ipsilateral, but not contralateral, ARC of the POMC-Cre; eGFP mice injected with retroAAV-FLEX-tdTomato. Importantly, most tdTomato-positive cells were found in the lateral part of the ARC. Scale bar: 50 μm. **(F)** Images of fluorescence confocal microscopy showing retrogradely labeled ARC POMC neurons. Most tdTomato neurons co-expressed GFP in the POMC-Cre; eGFP mice injected with retroAAV-FLEX-tdTomato into the MeA (white arrow). Scale bar: 30 μm. **(G)** Schematic drawing showing the location of retrogradely identified cells (x) in the ARC. 3V, 3rd ventricle.

As it has been shown that the MeA exhibits dense α-MSH-positive fibers (Jacobowitz and O’Donohue, 1978), we first sought to determine if the MeA is innervated by ARC POMC neurons. To examine this possibility, we used a Cre-dependent anterograde viral tracer AAV1-FLEX-GFPsm (Viswanathan et al., 2015). This viral tracer was stereotaxically injected into the ARC of the POMC-Cre animals (Figure 1A). Three to four weeks after such viral injections, we conducted immunohistochemistry with an anti-GFP antibody. Under these experimental conditions, Cre-mediated recombination resulted in the expression of GFP in ARC POMC neurons (Figure 1A). Also, we observed abundant GFP-positive fibers and terminals in the MeA (Figure 1B), indicating that ARC^POMC^ neurons send axonal projections to the MeA. Double immunostaining with an anti-adrenocorticotropic hormone (ACTH) antibody revealed that GFP-positive fibers in the MeA were co-labeled with ACTH (Figure 1C), consistent with early studies describing the presence of α-MSH and ACTH-positive fibers in the MeA (Jacobowitz and O’Donohue, 1978; Watson et al., 1978).

We further examined this ARC^POMC^→MeA projection with a Cre-dependent retrograde virus retroAAV-FLEX-tdTomato. RetroAAV-FLEX-tdTomato viruses were stereotaxically injected into the MeA of the POMC-Cre; eGFP mice (Figure 1D). Under these experimental conditions, mice injected with these viral tracers displayed tdTomato-positive fibers in the MeA (Figure 1D), indicating that these are POMC fibers. We found that there were retrogradely labeled tdTomato-positive cells in the ipsilateral, but not contralateral, ARC (Figure 1E). Most tdTomato-positive cells in the ARC co-expressed GFP (Figure 1F), supporting the interpretation that these cells are MeA-projecting POMC neurons in the ARC. Interestingly, the retrogradely identified POMC cells were located mainly in the lateral part of the ARC (Figures 1E,G).

### ARC POMC Neurons Innervate ER-α-Expressing Neurons in the MeA

**Figure 2:**
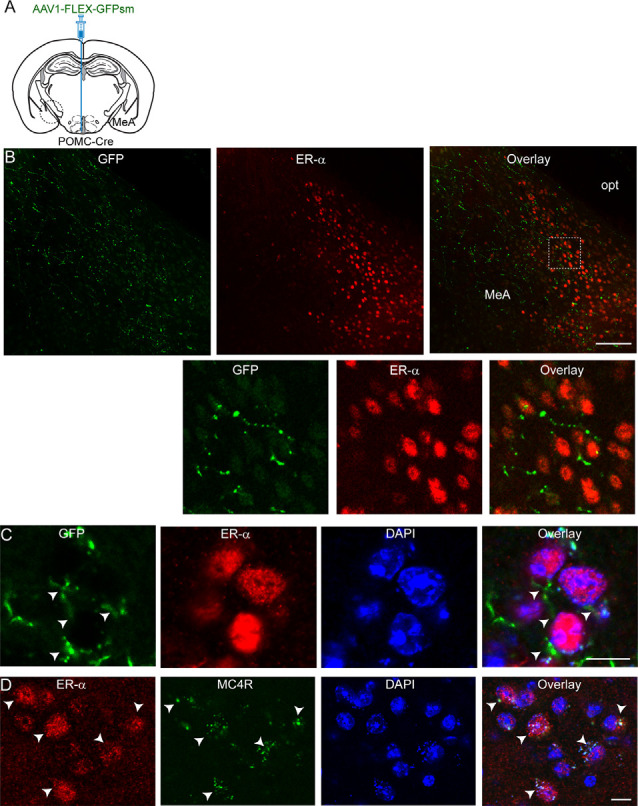
ARC POMC neurons project to ER-α-expressing cells in the MeA. **(A)** Schematic illustration of our experimental configurations. AAV1-FLEX-GFPsm was stereotaxically injected into the ARC of the POMC-Cre mice. **(B)** Images of fluorescence confocal microscopy showing that ARC POMC neurons send projections to ER-α-positive cells (top panel). GFP-positive fibers and axonal terminals were observed in the MeA where ER-α-positive cells were located. Scale bar: 100 μm. Bottom panel: images on the expanded scale (white square area in the top panel). **(C)** Images of fluorescence confocal microscopy showing that ER-α-positive cells receive POMC input from the ARC. GFP-positive fibers and axonal terminals made synaptic contacts with a subset of ER-α-positive cells (white arrowheads). Scale bar: 10 μm. **(D)** Images of fluorescence confocal microscopy showing co-expression of ER-α and MC4Rs (white arrows). Scale bar: 10 μm.

We then asked what neurons in the MeA receive melanocortinergic input from ARC POMC neurons. As the MeA contains abundant ER-α-expressing neurons (Merchenthaler et al., 2004; Xu et al., 2015; Saito et al., 2016) that control energy homeostasis (Xu et al., 2015), we examined if ER-α-expressing neurons is a downstream target of ARC POMC neurons by performing immunostaining with an anti-ER-α antibody. Anterograde AAV1-FLEX-GFPsm viruses were injected into the ARC of the POMC-Cre mice. Three to four weeks after viral injections, brain sections were double-stained with anti-ER-α and GFP antibodies (Figure 2A). As described in the prior studies (Merchenthaler et al., 2004; Xu et al., 2015; Saito et al., 2016), abundant ER-α-positive cells were detected in the MeA (Figure 2B). Double immunostaining revealed that GFP-positive fibers and terminals were close to ER-α-expressing neurons (Figures 2B,C).

We further investigated if these ER-α-positive cells express MC4Rs. Double immunostaining with anti-ER-α and anti-MC4R antibodies exhibited that ER-α-positive cells were positive for MC4Rs (Figure 2D). In our preparations, we found that about 90% of ER-α-positive cells coexpress MC4Rs (88.5 ± 1.7%; *n* = 220/249 ER-α-positive cells). These immunohistochemical results support the interpretation that ER-α-positive cells receiving direct synaptic input from ARC POMC neurons in the MeA express MC4Rs, consistent with prior studies showing SIM1 neurons in the MeA express ER-α as well as MC4Rs (Balthasar et al., 2005; Xu et al., 2015).

### Optogenetic Stimulation of the ARC^POMC→^MeA Projection Reduces Short-Term Food Intake

**Figure 3:**
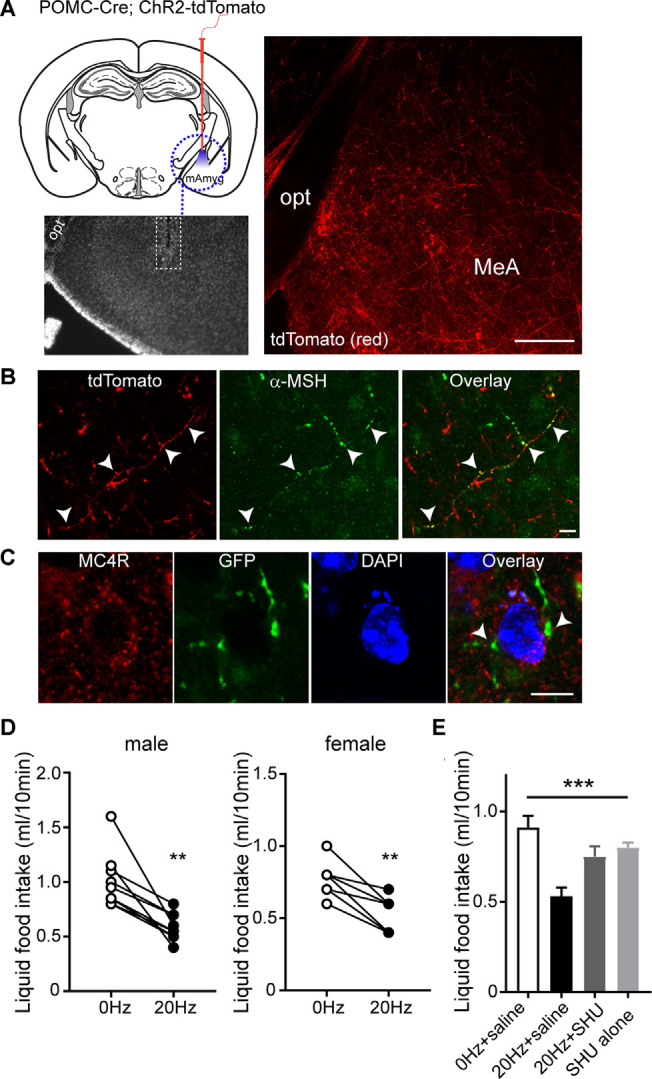
Activating the ARC^POMC^→MeA projection leads to an acute reduction in food intake.** (A)** Schematic drawing of our experimental configurations. A mono fiber-optic cannula was implanted into the MeA of the POMC-Cre; ChR2-tdTomato mice (top panel). The bottom panel shows the implantation site. tdTomato-positive fibers were observed in the MeA of POMC-Cre; ChR2-tdTomato mice. Scale bar: 200 μm. **(B)** Images of fluorescence confocal microscopy showing that tdTomato-positive fibers were positive for α-MSH in the MeA (white arrowheads). Scale bar: 10 μm. **(C)** Images of fluorescence confocal microscopy showing that MC4R-positive cells receive POMC input from the ARC. GFP-positive fibers and axonal terminals made synaptic contacts with MC4R-positive cells (white arrowheads). Scale bar: 10 μm. **(D)** Pooled data from nine male and seven female mice. Twenty hertz stimulation of the ARC^POMC^→MeA pathway significantly reduced liquid food intake (males, 1.0 ± 0.08 ml vs. 0.6 ± 0.04 ml, **p < 0.01, *n* = 9 mice; females, 0.8 ± 0.05 ml vs. 0.5 ± 0.05 ml, **p < 0.01, *n* = 7 mice). **(E)** Plot showing blockade of the effect of optogenetic stimulation of the ARC^POMC^→MeA pathway by the MC4R antagonist SHU9119 [1 mg/kg, *n* = 5 mice, SHU9119 alone, *n* = 8 mice, Treatment (between groups), *F*_(3,9)_ = 10.3, ***p < 0.001, 0 Hz + saline vs. 20 Hz + saline, *p* < 0.001, 20 Hz + saline vs. 20 Hz + SHU9119, *p* < 0.05, 20 Hz + saline vs. SHU9119 alone, *p* < 0.01].

As both MC4Rs and ER-α in the MeA play a role in the control of feeding (Liu et al., 2013; Xu et al., 2015), we investigated the effect of activation of the ARC^POMC^→MeA projection on feeding. To selectively stimulate POMC fibers in the MeA, we expressed light-activated proteins in POMC neurons by crossbreeding the POMC-Cre mice with the floxed-stop channelrhodopsin-2 (ChR2)-tdTomato mice (Figure 3A). In this mouse model, the MeA exhibited tdTomato-positive fibers and some tdTomato-positive fibers in the MeA were positive for α-MSH, further supporting the presence of the ARC^POMC^→MeA pathway (Figures 3A,B).

We next examined if the neurons receiving POMC input in the MeA express MC4Rs. We microinjected AAV1-FLEX-GFPsm to the ARC of POMC-Cre mice. In the MeA of POMC-Cre mice injected with these viruses, we found that GFP-positive fibers and terminals were close to MC4R-expressing cells (Figure 3C), suggesting that MC4R-expressing cells are a downstream target of ARC POMC neurons.

To measure short-term food intake, mice were mildly fasted from 12 PM to 6 PM and then given liquid food. Under these experimental conditions, ChR2-expressing POMC fibers in the MeA were illuminated with light 30 min before liquid food presentation. Bursts of light pulses (20 Hz) were applied for 1 s followed by a 3 s break that repeats continuously for 40 min as described in our recent studies (Jeong et al., 2015, 2017; Jeong et al., 2018a,b). We found that activating the ARC^POMC^→MeA pathway significantly reduced short-term liquid food intake in both male and female mice (Figure 3D).

We examined if the anorexigenic effect of optogenetic stimulation of the ARC^POMC^→MeA pathway is due in part to MC4R activation. We intraperitoneally injected the melanocortin 3/4 receptor antagonist SHU9119 30 min before optogenetic stimulation of the ARC^POMC^→MeA pathway. SHU9119 alone did not change liquid food intake (Figure 3E). Optogenetic stimulation of POMC fibers in the MeA did not affect liquid food intake in mice treated with SHU9119 (Figure 3E). Therefore, our results support the interpretation that the ARC^POMC^→MeA projection contributes to the control of short-term feeding, and ER-α- and MC4R-expressing cells are a potential downstream target of the pathway.

## Discussion

In the present study, we provide physiological evidence that in addition to the ARC^POMC^→PVH and ARC^POMC^→DMH^NPY^ circuits within the hypothalamus (Balthasar et al., 2005; Shah et al., 2014; Trotta et al., 2020), the ARC^POMC^→MeA pathway is another important neural circuit that mediates MC4R-mediated anorexic effects. We found that ARC POMC neurons sent axonal projections to the MeA. Interestingly, the projection neurons were located mainly in the lateral part of the ARC. ER-α-positive neurons as well as MC4R-expressing neurons in the MeA received direct synaptic input from ARC POMC neurons. A subset of ER-α-expressing cells was positive for MC4Rs, indicating that these ER-α- and MC4R-positive neurons were a downstream target of this projection. Optogenetic activation of the ARC^POMC^→MeA pathway caused a reduction in short-term liquid food intake in both female and male mice. This anorectic effect was blocked by the MC4R antagonist. As stress increases the activity of ARC POMC neurons (Liu et al., 2007; Qu et al., 2020) and MC4Rs in the MeA contribute to stress-induced behavior and hypophagia (Liu et al., 2013), the ARC^POMC^→MeA pathway that we identified may contribute to stress-induced short-term hypophagia.

Accumulating evidence has demonstrated heterogeneity of ARC POMC neurons (Parton et al., 2007; Hentges et al., 2009; Williams et al., 2010; Sohn et al., 2011; Jarvie and Hentges, 2012; Shi et al., 2013; Qiu et al., 2014; Dodd et al., 2018; Jones et al., 2019; Trotta et al., 2020). ARC POMC neurons are neurochemically, anatomically, and functionally heterogeneous (Parton et al., 2007; Hentges et al., 2009; Williams et al., 2010; Sohn et al., 2011; Jarvie and Hentges, 2012; Shi et al., 2013; Qiu et al., 2014; Dodd et al., 2018; Wei et al., 2018; Jones et al., 2019; Trotta et al., 2020). It has been recently shown that re-expression of the *Pomc* gene in ARC glutamatergic neurons in ARC specific *Pomc* KO mice completely normalizes body weight and body composition (Jones et al., 2019), although it is unknown if these effects are attributed to decreased food intake or increased energy expenditure, or both. Interestingly, POMC rescues exclusively in ARC GABAergic neurons are also able to normalize food intake and significantly reduce body weight. This anorectic effect is mediated by the ARC^POMC^→DMH^NPY^ projection (Trotta et al., 2020). The DMH is innervated largely by GABAergic POMC neurons and NPY expression in the DMH is inversely regulated by POMC expression in ARC GABAergic neurons (Trotta et al., 2020). These prior studies suggest that fast-acting neurotransmitters (i.e., glutamate and GABA) released from ARC POMC neurons are essential in regulating feeding and body weight.

Interestingly, it has been also described that ARC^POMC^ neurons do not release either glutamate or GABA to MC4R-expressing neurons in the PVH (Fenselau et al., 2017), a structure that relays information from the ARC to other brain areas including the lateral parabrachial nucleus. Instead, α-MSH released from ARC^POMC^ neurons post-synaptically regulates the strength of glutamatergic transmission in the PVN (Fenselau et al., 2017). This short-term synaptic plasticity by α-MSH appears to play a role in reducing feeding. In contrast to these well-known anorectic effects of α-MSH, it has been documented that activation of ARC POMC neurons can promote feeding by releasing β-endorphin (Koch et al., 2015; Wei et al., 2018). Therefore, it seems likely that both distinct axonal projections and neurotransmitters/neuropeptides released from ARC POMC neurons determine the net balance between anorexigenic melanocortin POMC and orexigenic opioid POMC neurons as suggested in our prior studies (Lee et al., 2015; Jeong et al., 2018b).

The MeA highly expresses MC4Rs (Kishi et al., 2003; Liu et al., 2003). Restoration of MC4Rs in the PVH as well as in the MeA in MC4R-deficient mice reduces food intake and body weight without changing energy expenditure compared with the loxTB MC4R mice (Balthasar et al., 2005). However, selective re-expression of MC4Rs in the MeA does not affect the body weight gain in mice lacking MC4Rs, suggesting that the PVH is the major site through which MC4Rs restrain feeding (Shah et al., 2014). However, the MeA receives synaptic input from ARC^AgRP^ neurons (Padilla et al., 2016). Stimulation of the ARC^AgRP^→MeA pathway drives feeding *via* inhibition of NPY 1 receptor-expressing neurons in the MeA (Padilla et al., 2016), suggesting that the central melanocortin system can control feeding *via* the neurons in the MeA. In our present study, we found that ARC^POMC^ neurons sent axonal projections to ER-α-expressing neurons in the MeA. The neurons receiving melanocortinergic input also expressed MC4Rs. These results suggest that the neurons receiving POMC input would be SIM1 neurons as SIM1 neurons express both receptors (Balthasar et al., 2005; Xu et al., 2015). Importantly, optogenetic activation of this pathway was able to acutely reduce food intake. Therefore, in addition to the PVH (Balthasar et al., 2005; Fenselau et al., 2017) and the DMH (Trotta et al., 2020), the MeA appears to be an important downstream target of both orexigenic AgRP and anorexigenic POMC neurons in the ARC.

The MeA is a brain region implicated in innate social behavior such as responses to a sexual partner, an aggressive intruder, or a predator (Cushing et al., 2008; Sano et al., 2016) and is critical for the processing of emotional stress (Ebner et al., 2004; Liu et al., 2013). It is known that fear and stress actively suppress eating. Chronic stress results in reduced body weight and anorexia that is associated with increased ARC POMC neuron activity (Qu et al., 2020). This anorectic effect in response to stress appears to be mediated through inhibition by opioids of dopamine-expressing neurons in the ventral tegmental area (VTA; Qu et al., 2020). As the MeA can respond to stress, it is plausible that the ARC^POMC^→MeA circuit that we identified would also contribute to stress-induced hypophagia. As stress promotes the activity of ARC POMC neurons (Liu et al., 2007; Qu et al., 2020), the increased POMC neuron activity would release α-MSH into the MeA and in turn activate ER-α-expressing neurons in the MeA, resulting in a short-term reduction in food intake. In this case, α-MSH and β-endorphin released by ARC POMC neurons would control feeding through the two distinct pathways (i.e., ARC^POMC^→MeA and ARC^POMC^→VTA^DA^). These findings further support the idea that both distinct neuronal projections and neurotransmitters/ neuropeptides released from ARC POMC neurons are crucial in determining the net balance between anorexigenic and orexigenic POMC neurons.

ER-α-expressing neurons in the MeA control metabolic homeostasis in both female and male mice (Xu et al., 2015) and in stress-induced pressor responses in females (Hinton et al., 2016). For instance, over-expression of ER-α in the MeA prevents DIO in male mice (Xu et al., 2015). Specific deletion of ER-α in the MeA causes obesity in male and female mice fed regular chow (Xu et al., 2015). These effects appear to be due in part to ER-α-induced activation of SIM1 neurons in the MeA (Xu et al., 2015). Activation of ER-α excites SIM1 neurons in the MeA. However, chemogenetic activation of SIM1 neurons changes neither food intake nor body temperature (Xu et al., 2015). These findings are somewhat inconsistent with prior findings (Xu et al., 2017; Estrada et al., 2018). Both local administration of estrogen to the MeA and subcutaneous estradiol treatment abolishes hyperphagia and body weight gain in ovariectomized female rodents (Xu et al., 2017; Estrada et al., 2018), suggesting that ER-α-expressing neurons in the MeA may have the ability to control feeding at least in female animals. Indeed, our present results support this possibility. Stimulation of the ARC^POMC^→MeA pathway significantly reduced feeding that is consistent with the feeding-regulating effects of MC4Rs in the MeA (Liu et al., 2013). Like melanocortinergic local circuits such as ARC^POMC^→PVH and ARC^POMC^→DMH^NPY^ neural circuits, this extrahypothalamic ARC^POMC^→MeA neural circuit would play a critical role in regulating feeding and possibly stress-mediated anorexia.

## Data Availability Statement

The raw data supporting the conclusions of this article will be made available by the authors, without undue reservation.

## Ethics Statement

The animal study was reviewed and approved by Institutional Animal Care Research Advisory Committee of the Albert Einstein College of Medicine.

## Author Contributions

EK performed the immunocytochemistry. Y-HJ designed the research, performed viral injection, immunocytochemistry, optogenetics, analyzed the data, and wrote the manuscript. All authors contributed to the article and approved the submitted version.

## Conflict of Interest

The authors declare that the research was conducted in the absence of any commercial or financial relationships that could be construed as a potential conflict of interest.
